# Yin Yang 1 contributes to gastric carcinogenesis and its nuclear expression correlates with shorter survival in patients with early stage gastric adenocarcinoma

**DOI:** 10.1186/1479-5876-12-80

**Published:** 2014-03-28

**Authors:** Wei Kang, Joanna HM Tong, Anthony WH Chan, Junhong Zhao, Yujuan Dong, Shiyan Wang, Weiqin Yang, Frankie MC Sin, Simon SM Ng, Jun Yu, Alfred SL Cheng, Ka Fai To

**Affiliations:** 1 Department of Anatomical and Cellular Pathology, State Key Laboratory in Oncology in South China, Prince of Wales Hospital, The Chinese University of Hong Kong, Hong Kong, SAR, PR China; 2 Institute of Digestive Disease, Partner State Key Laboratory of Digestive Disease, The Chinese University of Hong Kong, Hong Kong, SAR, PR China; 3 Li Ka Shing Institute of Health Science, Sir Y.K. Pao Cancer Center, The Chinese University of Hong Kong, Hong Kong, SAR, PR China; 4 Shenzhen Research Institute, The Chinese University of Hong Kong, Shenzhen, PR China; 5 Department of Medicine and Therapeutics, The Chinese University of Hong Kong, Hong Kong, PR China; 6 Department of Surgery, The Chinese University of Hong Kong, Hong Kong, PR China; 7 School of Biomedical Sciences, The Chinese University of Hong Kong, Hong Kong, PR China

**Keywords:** Yin Yang 1, Gastric adenocarcinoma, Prognostic marker, Oncogenic function

## Abstract

**Background:**

Yin Yang 1 (YY1) is a transcription factor that regulates diverse biological processes and increasing recognized to have important roles in carcinogenesis. The function and clinical significance of YY1 in gastric adenocarcinoma (GAC) have not been elucidated.

**Methods:**

In this study, the functional role of YY1 in gastric cancer was investigated by MTT proliferation assays, monolayer colony formation, cell cycle analysis, signaling pathway analysis, Western blot analysis and *in vivo* study through YY1 knockdown or overexpression. Immunohistochemical study with YY1 antibody was performed on tissue microarray consisting of 247 clinical GAC samples. The clinical correlation and prognosis significance were evaluated.

**Results:**

YY1 expression was up-regulated in gastric cancer cell lines and primary gastric cancers. Knocking down YY1 by siYY1 inhibited cell growth, inducing G1 phase accumulation and apoptosis. Ectopic YY1 expression enhanced cell proliferation *in vitro* and *in vivo*. Knocking down YY1 in gastric cancer cells suppressed proliferation by inhibiting Wnt/*β*-catenin pathway, whereas its overexpression exerted oncogenic property by activating Wnt/*β*-catenin pathway. In primary GAC samples, YY1 nuclear expression correlated with shorter survival and predicted poor prognosis in early stage GACs.

**Conclusion:**

Our data demonstrated that YY1 contributes to gastric carcinogenesis in gastric cancer. In early stage GACs YY1 might serve as a poor prognostic marker and possibly as a potential therapeutic target.

## Background

Gastric adenocarcinoma (GAC) is one of the most common malignancies in the world, with high rates of incidence in countries such as China, Japan, and South Korea [1]. Currently, GAC is one of the leading causes of cancer-related death worldwide, accounting for approximately 740,000 cases of cancer-related death annually [2]. While various factors such as *H. pylori* infection, genetic, epigenetic and molecular alterations affecting signaling pathways as well as genetic instability have been implicated in gastric tumorigenesis, the mechanisms of GAC pathogenesis are still largely unknown [3].

Yin Yang 1 (YY1) is a ubiquitously distributed transcription factor belonging to the Gli-Kruepple class of Zinc-finger proteins [4]. YY1 has diverse and complex biological functions and is involved in both repression and activation of numerous genes that play essential roles in a multitude of biological processes [5]. For example, YY1 has been shown to positively regulate several oncogenes, including c-Fos [6], c-Myc [7] and ERBB2 [8,9]. On the other hand, YY1 has also been found to negatively regulate several tumor suppressor genes such as p27 [10], p16 [11], p73 [12] and p53 [13].

YY1 was implicated in the carcinogenesis of a number of malignancies [14]. For example, by binding to the Snail 3′ enhancer, YY1 regulates the transcription of Snail in human melanoma cells [15]. In osteosarcoma, YY1 appears to be responsible for the tumor cells’ ability to invade and metastasize [16,17], and overexpression of YY1 in the primary site of osteosarcoma has shown to be associated with increased occurrence of metastasis and poor clinical outcome [18]. By affecting cell cycle and cellular motility, YY1 is involved in the transformation of non-neoplastic B cells to high grade B cell lymphomas [19]. In prostate cancer, YY1 physically interacts with androgen receptor (AR), which is required for the optimal transcriptional activity of AR in promoting the transcription of the prostate-specific antigen (PSA), a protein enhancing cell migration and metastasis [20]. YY1 promotes the expression of miR-190, a microRNA that is up-regulated in hepatic and pancreatic cancers and may play a role in AKT activation thus promotes growth factor-mediated cell survival [21,22].

In contrast, YY1 might serve as a tumor suppressor gene in several cancer types. In breast cancer, for instance, YY1 positively regulates the expression of breast cancer-associated gene 1 (BRCA1) [23] and heterochromatin protein 1 (HP1) [24]. YY1 also enhances the tumor suppressor DnaJ-like heat shock protein 40 (HLJ1) expression in a lung cancer cell model [25,26]. In follicular lymphoma, YY1 appears to act as a tumor suppressor and overexpression of YY1 is associated with favorable outcome with longer survival [27].

The expression and functional role of YY1 in gastric cancer is still unknown. In the current study, we aimed to investigate the functional role of YY1 in GAC and to examine its clinical significance in gastric cancer patients.

## Methods

### Cell line and cell culture

Ten gastric cancer cell lines (MKN28, KATOIII, MKN45, SNU16, SNU1, MKN7, MKN1, NCI-N87, AGS and MGC-803) were obtained from either the American Type Culture Collection (Rockville, MD) or RIKEN Cell Bank (Tsukuba, Japan), or received as a gift from Institute Digestive Disease of Prince Wales Hospital. These cell lines were grown in RPMI 1640 (GIBCO, Grand Island, NY) supplemented with 10% fetal bovine serum (FBS) (GIBCO, Grand Island, NY), 100 U/ml penicillin and 10 μg/ml streptomycin in a humidified atmosphere of 5% CO_2_ at 37°C.

### Patients and clinical GAC samples

The study was approved by Joint Chinese University of Hong Kong–New Territories East Cluster Clinical Research Ethics Committee, Hong Kong (CREC Ref. No. 2009.521) and all participants provided written informed consent for the collection of samples and subsequent analysis. A total of 264 GAC samples were retrieved from the tissue bank of Anatomical and Cellular Pathology, Prince of Wales Hospital, Hong Kong from 1998 to 2006. The 264 GAC samples were embedded into tissue microarray blocks. Another 10 pairs of primary tumors and adjacent non-tumorous tissues were collected intra-operatively from patients with GAC who had not received radiotherapy or chemotherapy prior to surgery. These specimens were immediately snap frozen at −80°C for molecular analysis.

### Immunohistochemistry and scoring

Immunohistochemistry was performed according to methods described previously [28]. Briefly, 4-μm-thick sections were obtained from formalin-fixed and paraffin-embedded specimens. After de-waxing in xylene and graded ethanol, sections were incubated in 3% H_2_O_2_ solution for 25 minutes to block endogenous peroxidase activities and then subjected to microwaving in EDTA buffer for antigen retrieval. Next, the tissue sections were incubated with the primary monoclonal YY1 antibodies (1:50, H-10, sc-7341, Santa Cruz Biotechnology, Dallas, TX) overnight at 4°C, and chromogen development was performed using the Envision system (DAKO Corporation, Glostrup, Denmark). The slides were independently scored by two investigators. The nuclear expression of YY1 was scored by estimating the proportion of tumor cells with positive nuclear staining into 4 different groups (0, none; 1+, <=10%; 2+, 10 to < =25%; 3+, >25%).

### RNA extraction and semiquantitative RT-PCR

Total RNA extraction was performed using TRIzol reagent (Invitrogen, Grand Island, NY) according to manufacturer’s instructions. RNA concentration was measured by NanoDrop 1000 (Thermo Fisher Scientific, Waltham, MA). High-Capacity cDNA Reverse Transcription Kits (Applied Biosystems, Grand Island, NY) were used for cDNA synthesis. For semiquantitative RT-PCR, 30-cycle touchdown PCR was applied for YY1 with sense primer GTCACCATGTGGTCCTCAGA and antisense primer CTGAGAGGTCAATGCCAGGT. The relative expression level was normalized with *β*-actin.

### Western blot analysis

Total protein was extracted from gastric cancer cell lines and paired primary tumors and non-tumorous tissues using RIPA lysis buffer with proteinase inhibitor. Protein concentration was measured by the method of Bradford (Bio-Rad, Hercules, CA). Twenty-microgram of protein mixed with 2 × SDS loading buffer were loaded on each lane, separated by 12% SDS-polyacrylamide gel electrophoresis. YY1 protein was then detected using anti-YY1 antibody (1:1000, H-10, sc-7341, Santa Cruz Biotechnology, Dallas, TX). Other antibodies applied included cleaved-PARP (Asp214) (1:1000, #9541, Cell Signaling, Danvers, MA), active-*β*-catenin (1:1000, #05-665, Millipore, Billerica, MA), *β*-catenin (1:10000, #610154, BD Transduction Laboratories, San Jose, CA), CCND1 (1:1000, #2926, Cell Signaling, Danvers, MA ), c-Myc (1:1000, #9402, Cell Signaling, Danvers, MA), anti-Mouse IgG-HRP (1:30000, #00049039, Dako, Glostrup, Denmark) and anti-Rabbit IgG-HRP (1:40000, #00028856, Dako, Glostrup, Denmark).

### Functional study assays *in vitro*

For cell proliferation assays, transfection of YY1 siRNA (SI00051912, QIAGEN, Valencia, CA), siCTNNB1 (SI02662478, QIAGEN, Valencia, CA) and scramble controls was performed by Lipofectamine 2000 Transfection Reagent (Invitrogen, Grand Island, NY). For the transfection of pcDNA3.1+ empty vector control and YY1, FuGENE HD transfection reagent (Roche, Basel, Switzerland) was employed. Cell proliferation was assessed using CellTiter 96 Non-Radioactive Cell Proliferation Assay (Promega, Madison, WI) according to manufacturer’s instructions. For colony formation assays in monolayer cultures, cells transfected with YY1 siRNA or scramble control were seeded into 6-well plates and cultured for 8 days. For YY1 overexpression colony formation assays, the transfected cells were selected by G418 (100 ng/ml) for 2 weeks. Cells were fixed with 70% ethanol for 15 minutes and stained with 2% crystal violet. Colonies with more than 50 cells per colony were counted. The experiments were repeated in triplicate wells. For cell cycle analysis, AGS, MKN28 and NCI-N87 cells were collected 24 hours following transfection in 6-well plates. Before transfection with siYY1, the cells underwent serum-free starvation for 12 hours for synchronization. Cells were harvested using cold PBS and fixed in 70% cold ethanol overnight at 4°C and treated with 1 ng/ml RNase A for 10 minutes at 37°C. Cellular DNA was stained with 15 ng/ml propidium iodide (PI) for 30 minutes at 37°C in the dark. The cells then were sorted by FACS Calibur Flow Cytometer (Becton Dickinson, San Diego, CA) and cell-cycle profiles were determined using the ModFitLT software (Becton Dickinson, San Diego, CA). The experiments were repeated twice.

### Signaling pathway analysis and validation

Cancer 10-pathway Reporter Luciferase Kit (QIAGEN, Valencia, CA) was employed to investigate the possible signaling pathways in which YY1 might be involved in 4 gastric cancer cell lines, AGS, MKN28, NCI-N87 and MGC-803. These cell lines were transfected with siYY1 and seeded in the Kit plate for luciferase activity detection. The Wnt/*β*-catenin signaling pathway was validated by TOPflash (reporter plasmid containing multiple copies of wild-type Tcf-binding sites) luciferase assays.

### *In vivo* tumorigenicity study

For YY1 knockdown *in vivo* study, MKN45 cells were transfected with empty vector (pBABE) and with shYY1. After puromycin selection, the cells (1 × 10^6^ cells suspended in 0.1 ml PBS) were injected subcutaneously into the dorsal flank of eight 4-week-old male Balb/c nude mice (shYY1 on the right side and the negative control cells on the left). Tumor diameter was measured and documented every 3 days until the tumor reached 10 mm in diameter. For YY1 overexpression *in vivo* study, MKN45 was transfected with empty vector (pcDNA3.1+) and with YY1. After G418 selection, the pool with stable YY1 overexpression and their control counterparts were injected subcutaneously into the dorsal flank of eight Balb/c nude mice (YY1 on the right side and the empty vector control cells on the left). Tumor diameter was measured and documented every 2 days until the tumors reached 10 mm in diameter. The mice were sacrificed and xenografts were removed for further validation. Tumor volume (mm^3^) was estimated by measuring the longest and shortest diameter of the tumor and calculated using the following formula: volume = (shortest diameter)^2^ × (longest diameter) × 0.5. All animal handling and experimental procedures were approved by the Animal Ethics Committee of the Chinese University of Hong Kong.

### Statistical analysis

The Mann–Whitney U test was used to compare the difference in biological behavior between siYY1 knockdown cells and scramble siRNA transfected cells. Correlations between YY1 nuclear stain and clinicopathologic parameters were assessed by the non-parametric Spearman’s rho rank test. The Kaplan-Meier method was used to estimate the survival rates for each variable. The equivalences of the survival curves were tested by log-rank statistics. For those variables being statistically significant found in the univariate survival analysis (*p* < 0.05), the Cox proportional hazards model with the likelihood ratio statistics was employed to further evaluate them for multivariate survival analysis. All statistical analyses were carried out using the statistical program SPSS version 16.0. A two-tailed *p*-value of < 0.05 was regarded as statistically significant.

## Results

### Up-regulation of YY1 in gastric cancer cell lines and primary GACs samples

**Figure 1:**
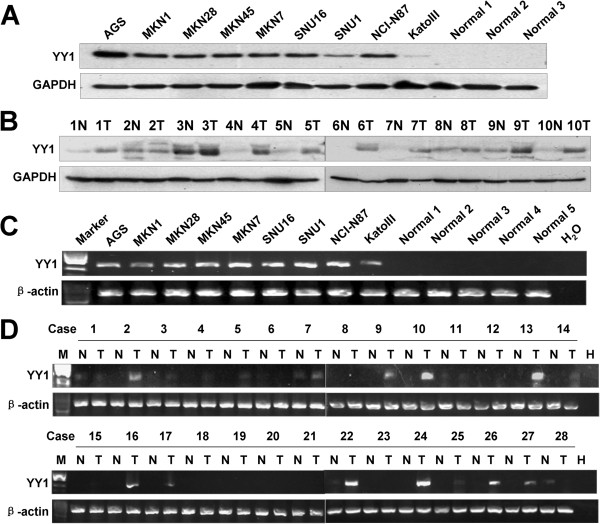
**YY1 expression in gastric cancer cell lines and clinical GAC samples. (A)** YY1 protein expression in 9 gastric cancer cell lines and 3 normal gastric tissues. **(B)** Western blot of YY1 in 10 pairs of gastric tumors (T) and the corresponding non-tumorous mucosa (N). **(C)** The expression of YY1 mRNA in 9 gastric cancer cell lines and 5 normal gastric tissues. **(D)** YY1 mRNA expression in 28 paired primary GACs (N, adjacent non-tumorous tissue; T, tumor tissue; H, water as negative control).

Up-regulation of YY1 protein was detected in 9 gastric cancer cell lines by Western blot analysis (Figure 1A). In contrast, the 3 non-neoplastic gastric tissue samples showed no evidence of YY1 protein. In addition, up-regulated YY1 protein expression was observed in 9 out of 10 primary GACs but not seen in any of the adjacent non-tumorous gastric tissues (Figure 1B). YY1 mRNA also showed positive expression in 9 gastric cancer cell lines, but there was no obvious expression in 5 normal gastric tissues (Figure 1C). In 28 paired GAC cDNAs, 15 cases showed strong expression of YY1 in tumor tissue compared with adjacent non-tumorous tissue (Figure 1D).

### YY1 knockdown has anti-oncogenic effect in GAC cells *in vitro*

**Figure 2:**
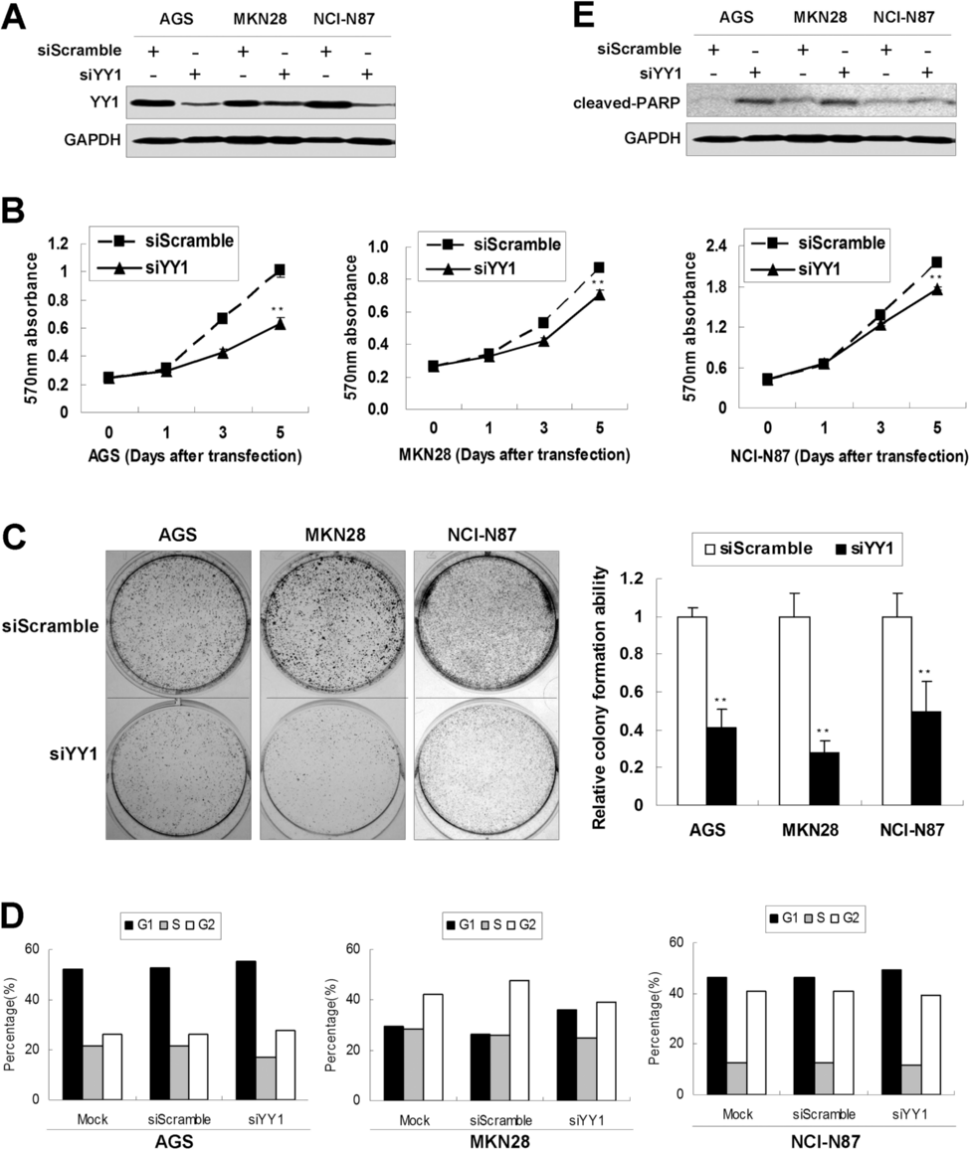
**YY1 knockdown inhibited proliferation in gastric cancer cell lines. (A)** Transfection with YY1 siRNA decreased YY1 protein expression in AGS, MKN28 and NCI-N87 cells. **(B)** 5-day MTT assays revealed YY1 siRNA suppressed gastric cancer cell proliferation (**, *p* < 0.001). **(C)** Monolayer colony formation assays suggested transfection with siYY1 reduced monolayer colony formation to 41.1%, 27.8% and 49.7% in AGS, MKN28 and NCI-N87 cells, respectively (**, *p* < 0.001). The experiments were done in triplicates. **(D)** Flow cytometry analysis revealed the accumulation of cells in G1 phase 24 hours after siYY1 treatment. Representative data from two independent experiments was shown. **(E)** Western blot of cleaved-PARP after siYY1 treatment in AGS, MKN28 and NCI-N87 cells.

YY1 protein was knocked down in AGS, MKN28 and NCI-N87 cells by siRNA mediated degradation (Figure 2A). A significantly decreased cellular proliferation was observed in these cell lines when compared with scramble siRNA groups (*p* < 0.001, Figure 2B). Monolayer colony formation assay indicated that YY1 knockdown significantly reduced colony formation in these cell lines (*p* < 0.001, Figure 2C). The transfectants were then analyzed for cell cycle parameters using flow cytometry. Twenty-four hours after transfection, accumulation of G1 cells increased from 52.6% to 55.3% in AGS, from 26.2% to 36.0% in MKN28, and from 46.6% to 49.4% in NCI-N87 (Figure 2D). The same trends were observed in a repeated set of experiments. Moreover, siYY1 induced late apoptosis, represented by an increase of cleaved-PARP, in three gastric cancer cell lines AGS, MKN28 and NCI-N87 (Figure 2E).

### YY1 knockdown suppresses Wnt/β-catenin signaling pathway and inhibits tumor growth *in vivo*

**Figure 3:**
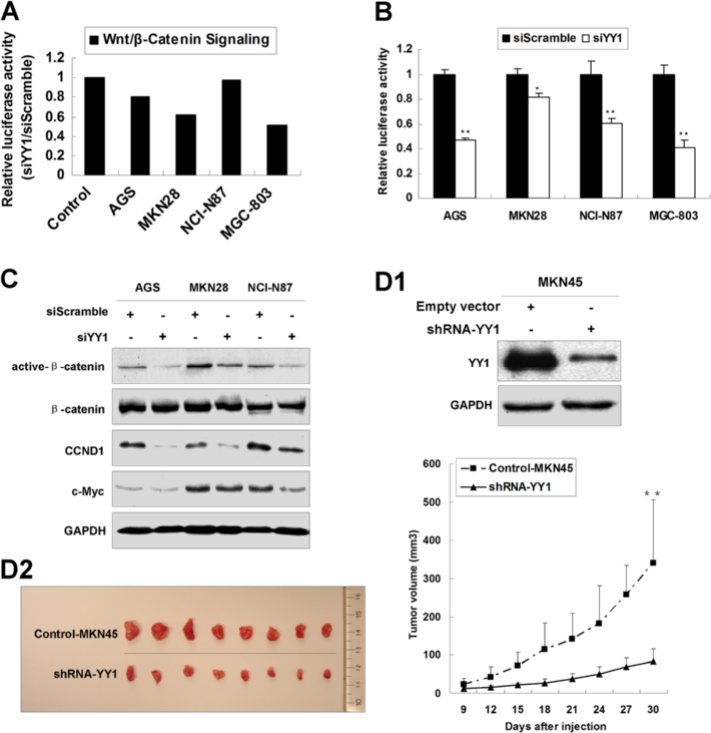
**YY1 knockdown suppressed Wnt/*β*-catenin signaling pathway and inhibits tumor growth *in vivo. *(A)** Relative luciferase activity of Wnt/*β*-catenin signaling pathway in 4 gastric cancer cell lines after siYY1 treatment. **(B)** Relative luciferase activity of TOPflash in AGS, MKN28, NCI-N87 and MGC-803 cells with siYY1 transfection (*, *p* < 0.05; **, *p* < 0.001). **(C)** Western blot of active-*β*-catenin, total *β*-catenin, CCND1 and c-Myc after siYY1 transfection in AGS, MKN28 and NCI-N87 cells. **(D1)** Western blot of YY1 expression in stable clones of shYY1-MKN45 and the vector control. **(D2)** shYY1-MKN45 formed smaller xenograft tumors than pBABE-MKN45 30 days after subcutaneous injection (**, *p* < 0.001).

By using Cancer 10-pathway Reporter Luciferase Kit, we found that siYY1 suppressed Wnt/*β*-catenin signaling pathway in AGS, MKN28 and MGC-803 cell lines (Figure 3A). We subsequently used TOPflash luciferase assays to confirm that Wnt/*β*-catenin signaling pathway was indeed inhibited by siYY1 in AGS, MKN28, NCI-N87 and MGC-803 cells (Figure 3B). Suppression of Wnt/*β*-catenin signaling pathway by siYY1 as indicated by decreased active-*β*-catenin, CCND1 and c-Myc was observed in siYY1 transfected AGS, MKN28 and NCI-N87 cells (Figure 3C).

The effect of shYY1 expression on *in vivo* growth of tumor was also studied. We first selected MKN45 clones which stably expressed shYY1 and validated the downregulated YY1 in shYY1 stable clones by Western blot (Figure 3D1). Then shYY1-MKN45 and vector control clones were injected into nude mice subcutaneously. The tumor growth in shYY1 expressing clones was significantly decreased compared with the vector control clones 30 days after injection (*p* < 0.001, Figure 3D2).

### YY1 overexpression enhances tumor growth both *in vitro* and *in vivo*

**Figure 4:**
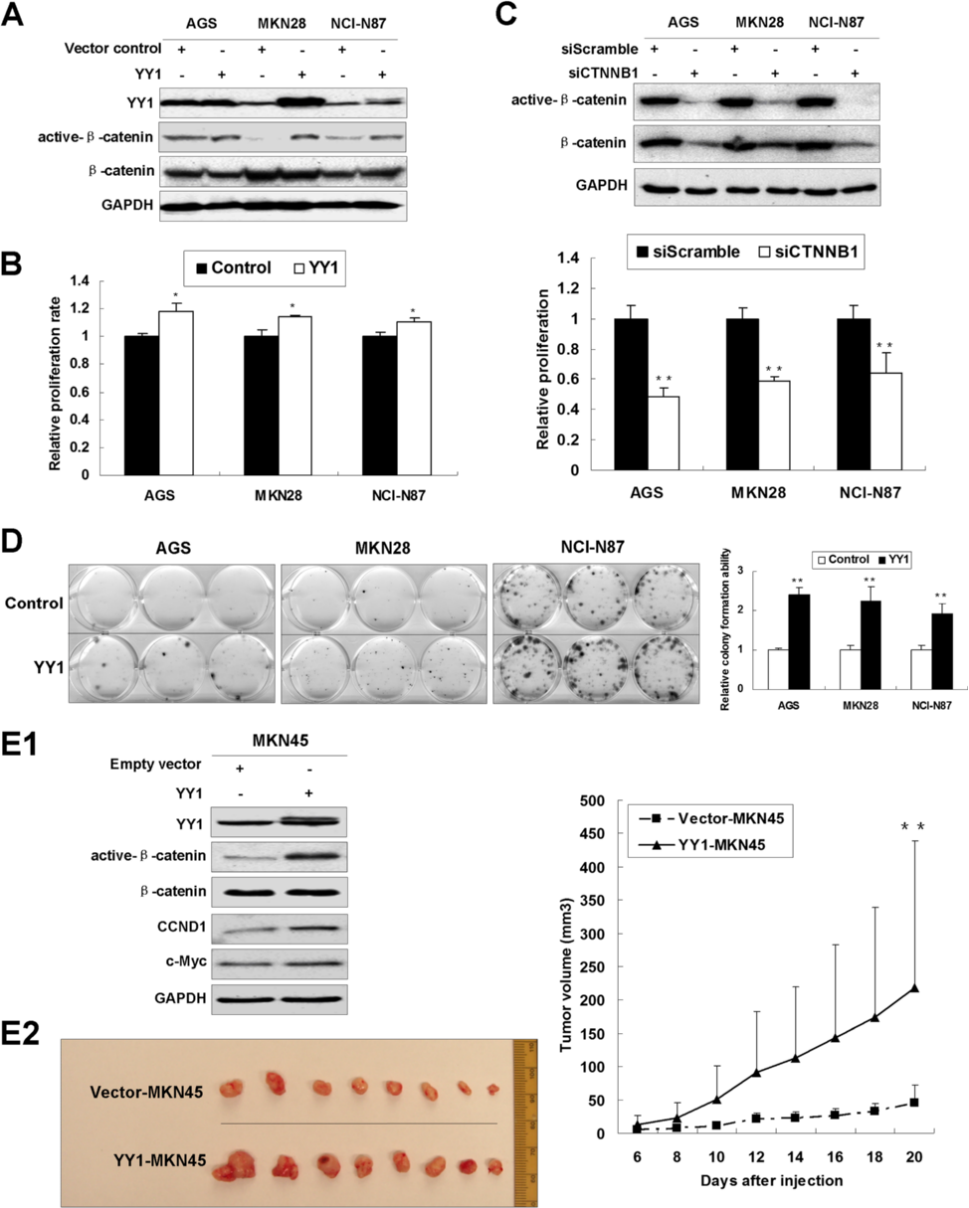
**YY1 ectopic expression enhanced tumor growth both *in vitro *and *in vivo*. (A)** Western blot of YY1, active-*β*-catenin and total *β*-catenin after YY1 exogenous overexpression in AGS, MKN28 and NCI-N87 cell lines. **(B)** 5-day MTT assays revealed YY1 enhanced gastric cancer cell proliferation (*, *p* < 0.05). **(C)** Upper: Western blot of active-*β*-catenin and total *β*-catenin under siCTNNB1 treatment. Lower: siCTNNB1 inhibits GAC cell proliferation (**, *p* < 0.001). **(D)** YY1 enhanced monolayer colony formation to 2.4, 2.3 and 1.9 folds in AGS, MKN28 and NCI-N87 cells, respectively, compared with vector controls (**, *p* < 0.001). The experiments were done in triplicates. **(E1)** YY1 ectopic expression activated Wnt/*β*-catenin signaling pathway in MKN45 cells by consecutively activating active-*β*-catenin, CCND1 and c-Myc. **(E2)** YY1-MKN45 formed bigger xenograft tumors than pcDNA3.1-MKN45 20 days after injection (**, *p* < 0.001).

To further investigate the YY1 functions in gastric cancer cells, we overexpressed YY1 into AGS, MKN28 and NCI-N87 cells. YY1 expression was first evaluated by Western blot after ectopic expression. Meanwhile active-*β*-catenin showed increased level (Figure 4A). YY1 overexpression increased cell proliferation in these three cell lines in 5-day MTT assays (*p* < 0.05, Figure 4B). To confirm the proliferation-promoting effect by YY1 through Wnt/*β*-catenin signaling pathway, siCTNNB1 (25 nM) was transfected into the cultured cells to knockdown Wnt pathway effector *β*-catenin. The *β*-catenin was effectively knocked down and the same grow suppressive effect was observed (Figure 4C), indicating that Wnt/*β*-catenin signaling pathway plays an important role in the YY1-induced tumor growth.

We further examined the effect of YY1 on monolayer colony formation. Gastric cancer cells over-expressing YY1 formed more and larger colonies (2.4, 2.3 and 1.9 folds in AGS, MKN28 and NCI-N87) in comparison to vector control groups, (*p* < 0.001, Figure 4D). The activated Wnt/*β*-catenin signaling pathway together with upregulation of its downstream effectors CCND1 and c-Myc were observed in YY1-MKN45 stable clones (Figure 4E1). The YY1-MKN45 stable clones were injected subcutaneously into the dorsal flank of 8 nude mice. Twenty days later, YY1 over-expressing clones formed larger xenografts than the control clones (*p < 0*.001, Figure 4E2).

### Nuclear expression of YY1 correlates with shorter survival time in patients with early stage GACs

**Figure 5:**
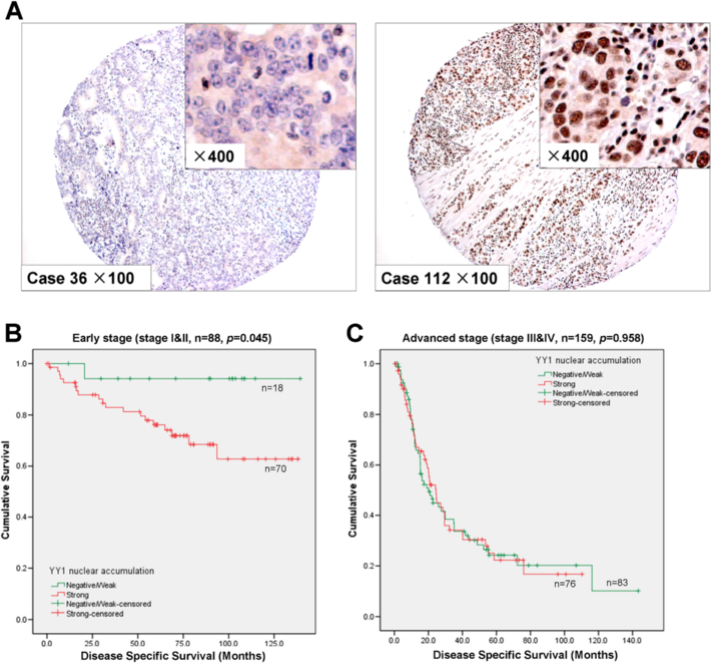
**YY1 nuclear expression correlated with poor survival in early stage gastric cancers. (A)** Representative figures of YY1 immunohistochemistry in primary GACs. Left, negative/low expression case; right, positive expression case (original magnification × 100, insertion × 400). **(B)** YY1 nuclear expression predicted poor prognosis in early stage GAC patients (stage I and II, *p* = 0.045). **(C)** YY1 nuclear expression did not associate with disease specific survival in advanced stage GACs (stage III and IV, *p* = 0.958)*.*

YY1 was predominantly expressed in the nuclei of tumor cells (Figure 5A; left, case with negative YY1 expression; right, case with strong YY1 expression). Of the 247 GAC samples, 98 cases (39.7%) had a YY1 nuclear score 0, 3 cases (1.2%) had a score 1+, 5 cases (2%) had a score of 2+, and 141 cases (57.1%) had a score of 3+. In total, 101 of 247 samples (40.9%) of gastric cancer cases had negative/weak (0 to 1+) YY1 expression, while 146 of 247 samples (59.1%) had strong YY1 expression (2+ to 3+, Additional file 1: Figure S1). No survival difference was observed between YY1 negative/weak and strong expression by univariate analysis (*p* = 0.341).

We further investigated the clinicopathologic correlation of YY1 by stratifying the patients into early stage (stage I and II, n = 88) and advanced stage (stage III and IV, n = 159). Table 1 summarized the correlation of YY1 with clinicopathologic parameters in early stage and advanced stage patients. In early stage patients, YY1 expression correlated with diffuse type gastric cancer (*p* = 0.031). Univariate analysis (Table 2) indicated that YY1 strong expression in early stage GACs associated with poor disease specific survival (*p* = 0.045, Figure 5B). Negative *H. pylori* infection also correlated with poor survival (*p* = 0.003). By multivariate Cox proportional hazards regression analysis (Table 2), only *H. pylori* infection was independently associated with disease specific survival (*p* = 0.007).

**Table 1:** **Correlation of YY1 nuclear expression with clinicopathologic features (significant *p*-value in bold and italic format)**

		**Early stage cases (stage I & II, n = 88)**			**Advanced stage cases (stage III & IV, n = 159)**		
		**YY1 nuclear expression**			**YY1 nuclear expression**		
		**Negative/weak**	**Strong**	***p-*** **value**	**Negative/weak**	**Strong**	***p*-value**
		**Number (%)**	**Number (%)**		**Number (%)**	**Number (%)**	
Sex	M	12 (20.3)	47 (79.7)	0.969	56 (52.3)	51 (47.7)	0.961
	F	6 (20.7)	23 (79.3)		27 (51.9)	25 (48.1)	
Age	<=60	6 (20.7)	23 (79.3)	0.969	39 (61.9)	24 (38.1)	0.053
	>60	12 (20.3)	47 (79.7)		44 (45.8)	52 (54.2)	
Type	Intestinal	17 (26.2)	48 (73.8)	***0.031***	47 (61.8)	29 (38.2)	***0.020***
	Diffuse	1 (4.5)	21 (95.5)		36 (43.4)	47 (56.6)	
Grade	1	2 (28.6)	5 (79.7)	0.866	1 (100.0)	0 (0.0)	***0.033***
	2	8 (20.0)	32 (80.0)		38 (64.4)	21 (35.6)	
	3	8 (20.0)	32 (80.0)		44 (44.4)	55 (55.6)	
Stage (T)	1	7 (21.2)	26 (78.8)	0.909	1 (50.0)	1 (50.0)	0.971
	2	10 (21.3)	37 (78.7)		13 (52.0)	12 (48.0)	
	3	1 (14.3)	6 (85.7)		65 (52.8)	58 (47.2)	
	4				4 (44.4)	5 (55.6)	
Stage (N)	0	13 (23.6)	42 (76.4)	0.623	1 (50.0)	1 (50.0)	0.841
	1	5 (16.1)	26 (83.9)		17 (53.1)	15 (46.9)	
	2	0 (0.0)	1 (100.0)		36 (48.6)	38 (51.4)	
	3				29 (56.9)	22 (43.1)	
Stage (M)	0	18 (20.5)	70 (79.5)		64 (52.0)	59 (48.0)	0.937
	1				19 (52.8)	17 (47.2)	
Lymph Node	0	13 (23.6)	42 (76.4)	0.374	1 (50.0)	1 (50.0)	0.950
	1	5 (15.6)	27 (84.4)		82 (52.2)	75 (47.8)	
*H. pylori*	Absence	7 (18.4)	31 (81.6)	0.743	36 (47.4)	40 (52.6)	0.417
	Presence	10 (21.3)	37 (78.7)		41 (53.9)	35 (46.1)	

**Table 2:** **Univariate and multivariate Cox regression analysis of clinicopathologic factors in patients with gastric adenocarcinoma (significant *p*-value in bold and italic format)**

	**Early stage cases (stage I & II, n = 88)**		**Advanced stage cases (stage III & IV, n = 159)**	
	**Univariate**	**Multivariate**	**Univariate**	**Multivariate**
Sex	0.900		0.318	
Age	0.120		***0.006***	***0.003***
Type	0.342		0.654	
Grade	0.594		0.747	
Stage (T)	0.974		0.088	
Stage (N)	0.656		***0.002***	***<0.001***
Stage (M)			***<0.001***	***<0.001***
*H. pylori*	***0.003***	***0.007***	0.611	
YY1	***0.045***	0.970	0.958	

In advanced stage GACs (Table 1), YY1 nuclear expression correlated with histology with diffuse component (*p* = 0.020), higher tumor grade (*p* = 0.033). However, YY1 expression could not predict survival (Figure 5C, *p* = 0.958) by univariate analysis. Old age (age > 60, *p* = 0.006), advanced N stage (*p* = 0.002) and advanced M stage (*p* < 0.001) also predicted poor prognosis. By multivariate Cox proportional hazards regression analysis, age, N stage and M stage remained the independent prognostic factors for patients with advanced gastric cancer.

## Discussion

YY1 serves either as a tumor suppressor gene or oncogene depending on the types of tumors it is expressed in [23,29-33]. The choice of its function and consequently the final outcome might be determined by multiple factors such as cell context, oncogenic stimulation or the regulation of its upstream pathways. As a transcription factor, YY1 binds the promoter of associated oncogenes and exert its oncogenic property [34]. In prostate cancer, YY1 has two binding sites within the prostate stem cell antigen (PSCA) promoter facilitating development of malignant human prostate cancer [30]. YY1 also reportedly activates the expression of human epidermal growth factor receptor 2 (ERBB2) [8,9], which is overexpressed in approximately 30% of breast cancers and correlates with poor prognosis. YY1 induces expression of cyclooxygenase-2 (COX-2), which is overexpressed in 40% of human invasive breast cancers and mediates bone metastasis [35]. In Burkitt lymphoma, YY1 binds to this HS3 enhancer and recruits CBP to this region, which increases the histone acetylation of the c-Myc promoter and activates c-Myc gene expression [36]. YY1 forms an active complex with hypoxia inducible factor (HIF) 1α to activate vascular endothelial growth factor (VEGF) gene expression [37,38]. In gastric cancer, we provide the first evidence that YY1 also plays an oncogenic role. This was indicated by YY1 overexpression enhanced cell proliferation, monolayer colony formation and xenograft growth whereas YY1 knockdown inhibited gastric cancer cell proliferation both *in vitro* and *in vivo*.

The current study also suggested that YY1 affected Wnt signaling cascades in gastric cancer cells. By using Cancer 10-pathway Reporter Luciferase Assay, we identified that siYY1 inhibited Wnt/*β*-catenin signaling pathway in gastric cancer cell lines. The finding was further validated by the TOPflash luciferase assays. Suppression of Wnt/*β*-catenin pathway by siYY1, as evidenced by decrease active-*β*-catenin, CCND1 and c-Myc level had been demonstrated. On the contrary, YY1 overexpression promotes Wnt/*β*-catenin pathway and up-regulates its targets. These data were in keeping with a recent finding that YY1 activates Wnt signaling pathway through activating *β*-catenin in colon cancer [39]. Using microarray analysis, Zhang et al. found that YY1 regulated the expression of a number of Wnt-associate genes. It is plausible to speculate that YY1 might promote the Wnt signaling pathway by suppressing Wnt antagonists/inhibitors, i.e. CSNK1A1, CTNNBIP1, SFRP1 and the deletion variant of LEF-1, and up-regulate Wnt initiators, i.e. CTNNB1, FZD4, Wnt1 and Wnt3a. Our data clearly indicated that YY1 acted to promote Wnt/*β*-catenin signaling in gastric carcinogenesis.

To determine the clinical relevance of YY1 in primary GACs, we examined the protein expression of YY1 in 247 clinical gastric cancer samples. The overexpression of YY1 was not correlated with TNM staging, this result was partly concordant with previous reports that there were no difference for the YY1 expression between primary tumor and metastatic samples [40]. Nevertheless, expression of YY1 associated with diffuse type histology both in early-stage and advanced-stage GACs, suggesting that YY1 might be involved in the occurrence and development of diffuse type GACs. Comparing with intestinal type GACs, diffuse type GACs are less related to atrophy or intestinal metaplasia, occur more often in younger patients and are associated with a poorer prognosis. Our findings supported that YY1 overexpression is involved in the carcinogenesis diffuse type GACs. In early stage (stage I and II) GACs, YY1 nuclear expression correlated with shorter survival and predicts poorer prognosis. This result was concordant with the clinical significance of YY1 in colon cancer [39].

## Conclusion

In conclusion, we provided both *in vitro* and *in vivo* evidence that YY1 contributes to gastric tumorigenesis through promoting cell survival in GAC cells. Functional studies demonstrated that downregulation of YY1 expression by siYY1 quenched its oncogenic properties by inhibiting cell growth, inducing G1 phase accumulation and apoptosis. YY1 overexpression enhanced cell proliferation by activating the Wnt/*β*-catenin signaling pathway. YY1 nuclear expression correlated with poor prognosis in patients with early stage GAC, suggesting that YY1 might potentially serve as a prognostic biomarker and a therapeutic target in gastric cancer.

## Competing interests

The authors declare that they have no competing interests.

## Authors’ contributions

WK, JHMT, AWHC, JHZ, YJD, SYW, WQY and FMCS carried out the experimental studies, interpreted the data, performed the statistical analysis. SSMN, JY, ASLC provided experimental materials. WK, JHMT and KFT contributed to the study design, manuscript drafting and provided fund for this study. All authors read and approved the final manuscript.

## Supplementary Material

Additional file 1: Figure S1 Representative figures of YY1 immunohistochemistry in 25 primary GACs with strong YY1 expression (2+ or 3+, original magnification × 100, insertion × 400). The up-regulated expression of YY1 mainly localized in the nuclei of the cancer cells. Click here for file
