# Supplementary figures and images for: Yin Yang 1 contributes to gastric carcinogenesis and its nuclear expression correlates with shorter survival in patients with early stage gastric adenocarcinoma
Source: J Transl Med. 2014 Mar 28;12:80. doi: 10.1186/1479-5876-12-80 (PMC3986816; doi:10.1186/1479-5876-12-80)

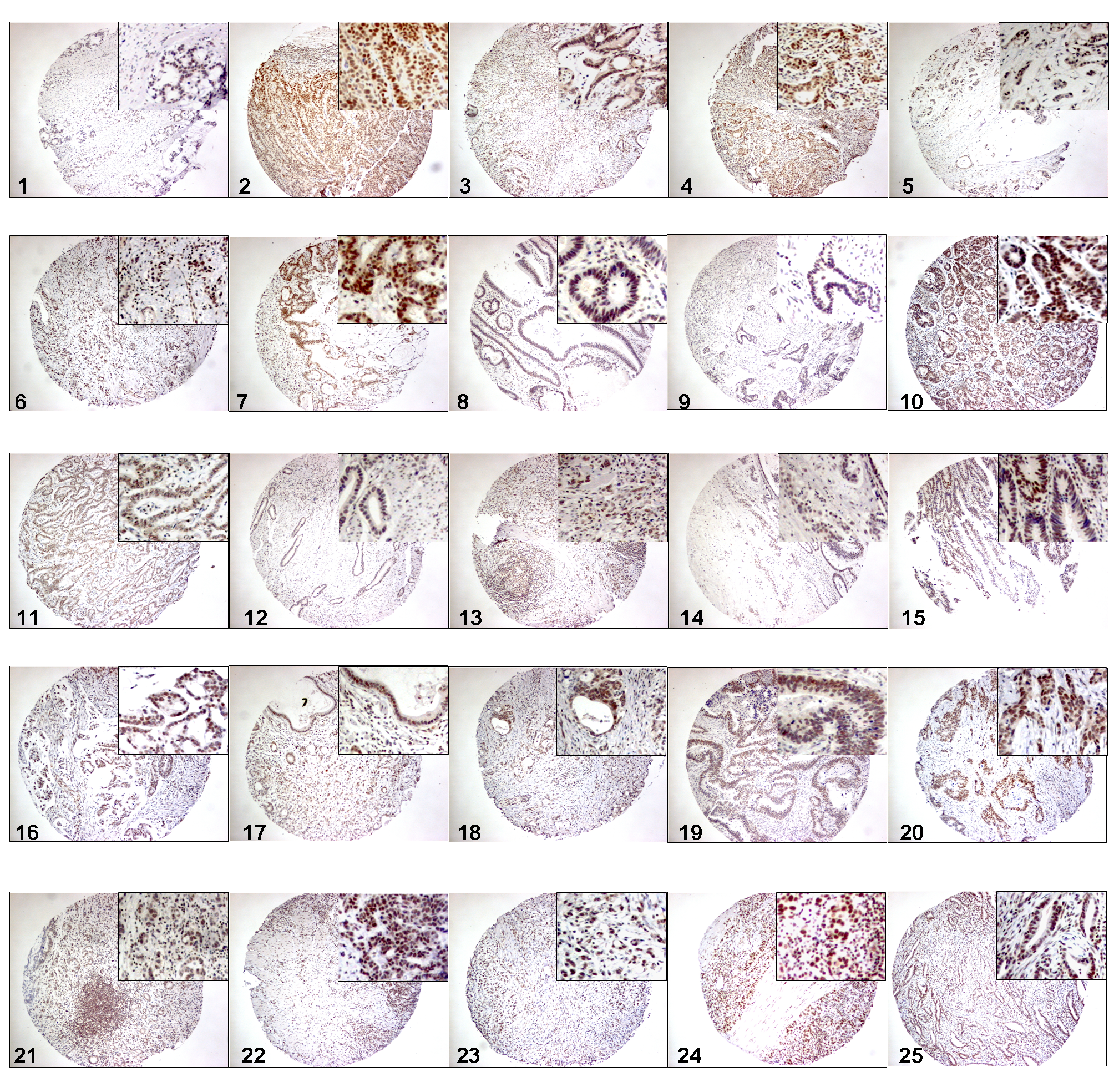

Supplement: Additional file 1: Figure S1 — Representative figures of YY1 immunohistochemistry in 25 primary GACs with strong YY1 expression (2+ or 3+, original magnification × 100, insertion × 400). The up-regulated expression of YY1 mainly localized in the nuclei of the cancer cells. [file 1479-5876-12-80-S1.tiff]
